# Hierarchical Sheet-on-Sheet ZnIn_2_S_4_/g-C_3_N_4_ Heterostructure with Highly Efficient Photocatalytic H_2_ production Based on Photoinduced Interfacial Charge Transfer

**DOI:** 10.1038/srep19221

**Published:** 2016-01-12

**Authors:** Zhenyi Zhang, Kuichao Liu, Zhiqing Feng, Yanan Bao, Bin Dong

**Affiliations:** 1 Key Laboratory of New Energy and Rare Earth Resource Utilization of State Ethnic Affairs Commission, School of Physics and Materials Engineering, Dalian Nationalities University, 18 Liaohe West Road, Dalian 116600, P. R. China

## Abstract

We have realized *in-situ* growth of ultrathin ZnIn_2_S_4_ nanosheets on the sheet-like g-C_3_N_4_ surfaces to construct a “sheet-on-sheet” hierarchical heterostructure. The as-synthesized ZnIn_2_S_4_/g-C_3_N_4_ heterojunction nanosheets exhibit remarkably enhancement on the photocatalytic activity for H_2_ production. This enhanced photoactivity is mainly attributed to the efficient interfacial transfer of photoinduced electrons and holes from g-C_3_N_4_ to ZnIn_2_S_4_ nanosheets, resulting in the decreased charge recombination on g-C_3_N_4_ nanosheets and the increased amount of photoinduced charge carriers in ZnIn_2_S_4_ nanosheets. Meanwhile, the increased surface-active-sites and extended light absorption of g-C_3_N_4_ nanosheets after the decoration of ZnIn_2_S_4_ nanosheets may also play a certain role for the enhancement of photocatalytic activity. Further investigations by the surface photovoltage spectroscopy and transient photoluminescence spectroscopy demonstrate that ZnIn_2_S_4_/g-C_3_N_4_ heterojunction nanosheets considerable boost the charge transfer efficiency, therefore improve the probability of photoinduced charge carriers to reach the photocatalysts surfaces for highly efficient H_2_ production.

Photocatalytic H_2_ production through water splitting or reduction has received great attention in recent years, since it offers an economical and environmentally friendly strategy to convert solar energy into preservable chemical fuels for mitigating the excessive consumption of non-sustainable fossil fuels such as coal, petroleum and natural gas^1^,^2^,^3^,^4^. To date, various kinds of UV- or visible-response semiconductors, such as TiO_2_, ZnO, Cu_2_O, CdS, and so forth, have been developed as photocatalysts for H_2_ production due to their suitable band potential and catalytic functions^5^,^6^,^7^,^8^,^9^,^10^. Besides these traditional semiconductor photocatalysts, a large number of semiconductive materials, including metal-organic frameworks (MOFs), polyoxometalate (POM), and metal free compounds, are also being introduced as potential candidates for the new generation of photocatalysts to fulfill the photocatalytic water reduction and oxidation^11^,^12^,^13^,^14^. Among them, the two-dimensional (2D) layered polymer, graphitic carbon nitride (g-C_3_N_4_), is considered as the most promising visible-light-active photocatalyst because of its unique electronic structure, high stability, nontoxic nature, and low cost^15^,^16^,^17^. However, the photocatalytic activity on H_2_ production over single-component g-C_3_N_4_ so far is unsatisfactory due to its limited surface areas, poor light-harvesting efficiency and fast recombination of photoinduced charge carriers^15^,^17^. To overcome the above drawbacks, much effort has been devoted to the construction of multi-component heterostructural photocatalysts through coupling other visible-active semiconductor with g-C_3_N_4_ nanosheets, in which the heterogeneous interfaces can effectively assist the photoinduced charge-carriers migration and hinder these charge-carriers recombination to enhance the photocatalytic efficiency^18^,^19^,^20^,^21^,^22^,^23^,^24^. Moreover, the surface structures and light-harvesting behavior can be also promoted through tailoring the secondary nanostructures of heterostructural photocatalysts^25^,^26^,^27^. Therefore, design and architecture of g-C_3_N_4_-based heterostructural photocatalysts with the matchable bandgap, desired component, and hierarchical nanostructures is still the hot topics in the field of solar-to-fuels conversion.

As an important ternary chalcogenide semiconductor, hexagonal phase ZnIn_2_S_4_ with 2D layered structure and narrow bandgap has been extensively investigated in photocatalysis, especially serving as the photosynergistic components in heterojunction photocatalysts to enhance the photocatalytic efficiency for H_2_ production^28^,^29^,^30^,^31^,^32^. In the viewpoint of band structure, the bandgap of ZnIn_2_S_4_ (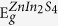 = ∼2.6 eV) is smaller than that of g-C_3_N_4_ (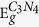 ~2.8 eV) while the conduction band (CB) of ZnIn_2_S_4_ (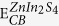 = −1.0 V) is higher than that of g-C_3_N_4_ (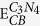 = −1.1 V)^15^,^17^,^28^,^33^. Accordingly, when integrating of ZnIn_2_S_4_ nanostructure with g-C_3_N_4_ nanosheets, a “type I” heterojunction would be formed in their interface, meaning that the CB and valence band (VB) positions of g-C_3_N_4_ straddle those of ZnIn_2_S_4_. As such, the ZnIn_2_S_4_ can be seemed as a “charge sink” to accept the photoinduced charge carriers from adjacent g-C_3_N_4_, leading to the improvement of charge separation on g-C_3_N_4_ and thereby enhancing its photocatalytic activity. On the other hand, the 2D sheet-like ZnIn_2_S_4_ nanostructures could be easily anchored onto the active or flexible 2D substrates, such as F-doped SnO_2_ (FTO) thin film and reduced graphene oxide (RGO) nanosheets, to form the “sheet-on-sheet” type heterostructure. This kind of hierarchical nanostructure usually exhibits a high surface area, strong light harvesting, and efficient charge mobility due to its unique structure advantages^25^,^26^,^29^,^31^,^33^,^34^. The above analysis implies that once the ZnIn_2_S_4_ nanosheets combine with g-C_3_N_4_ nanosheets, a significant enhancement on photocatalytic H_2_ production may be realized through synergistic promotion on the inner charge carriers and outer hierarchical structures. However, little effort has been donated to the synthesis of ZnIn_2_S_4_/g-C_3_N_4_ heterostructure toward the highly efficient photocatalytic H_2_ production. Herein, we report a novel kind of “sheet-on-sheet” heterostructure synthesized through *in-situ* growth of ultrathin ZnIn_2_S_4_ nanosheets onto g-C_3_N_4_ nanosheets surfaces. After introducing the ZnIn_2_S_4_ nanosheets, the specific surface area of g-C_3_N_4_ nanosheets is obviously promoted, resulting in providing the more active sites for the photoreaction. Furthermore, the intimate contacted interface between the ZnIn_2_S_4_ and g-C_3_N_4_ nanosheets facilitates the photoinduced charge-carriers transfer from g-C_3_N_4_ to ZnIn_2_S_4_ based on the heterojunction effect. By taking of the above features, the as-synthesized ZnIn_2_S_4_/g-C_3_N_4_ heterojunction nanosheets exhibit a significantly enhanced visible-light photocatlaytic H_2_ production performance as compared to the single component of ZnIn_2_S_4_ or g-C_3_N_4_ nanosheets.

## Results

**Figure 1:**
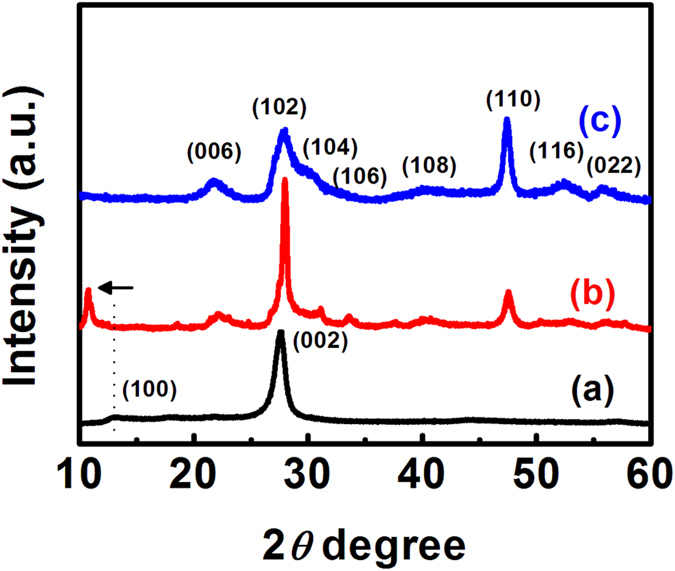
XRD patterns of the as-synthesized samples: (**a**) g-C_3_N_4_ nanosheets; (**b**) 15 wt% ZnIn_2_S_4_/g-C_3_N_4_ heterojunction nanosheets; (**c**) ZnIn_2_S_4_ nanosheets.

X-ray diffraction (XRD) patterns of the as-synthesized samples are shown in Fig. 1. Two pronounced diffraction peaks appear at 13.1° and 27.4° for g-C_3_N_4_ nanosheets, reflecting to the periodic structure of intra-planar tri-s-triazine packing as the (100) peak, and the interlayer stacking of conjugated aromatic structures as the (002) peak for graphitic materials, respectively^11^,^15^,^17^. The diffraction peaks of ZnIn_2_S_4_ nanosheets can be perfectly indexed as a pure hexagonal phase of ZnIn_2_S_4_ (JCPDS No. 65–2023)^28^,^29^,^31^. In the case of ZnIn_2_S_4_/g-C_3_N_4_ heterojunction nanosheets, the XRD pattern shows diffraction peaks of both ZnIn_2_S_4_ and g-C_3_N_4_ nanosheets, while the feature peaks of ZnIn_2_S_4_ (27.7°) and g-C_3_N_4_ (27.4°) are very close and overlap with each other. Note that the diffraction peaks of ZnIn_2_S_4_ nanosheets are very weak. This phenomenon may be ascribed to two reasons: (1) the ultrathin 2D nanostructure of ZnIn_2_S_4_, leading to the ultra-small size in its c-axis orientation^31^; (2) the low content of ZnIn_2_S_4_ component in the heterostructure^25^. Besides, after introducing the ZnIn_2_S_4_ by hydrothermal method, the (100) diffraction peak of g-C_3_N_4_ becomes more intense, and its position shifts toward the lower diffraction angle (10.8°) (Figure S1). This reveals that some metal ions from ZnIn_2_S_4_ surfaces may be connected with the g-C_3_N_4_ through the lone-pair electrons of nitrogen in the “nitrogen pots”, thus leading to enlarging the intra-planar separation of ordered tri-s-triazine packing^21^,^22^,^35^. Moreover, some weak peaks corresponded to the intermediates of thermal polymerized g-C_3_N_4_ are also detected on the XRD pattern of ZnIn_2_S_4_/g-C_3_N_4_ heterojunction nanosheets, implying that a small part of g-C_3_N_4_ nanosheets might be further exfoliated (or reduced) into the structural units of g-C_3_N_4_, such as melamine, ammeline, or tri-s-triazine units, due to the longer reaction time for hydrothermal growth of ZnIn_2_S_4_ onto g-C_3_N_4_ nanosheets^16^,^36^.

**Figure 2:**
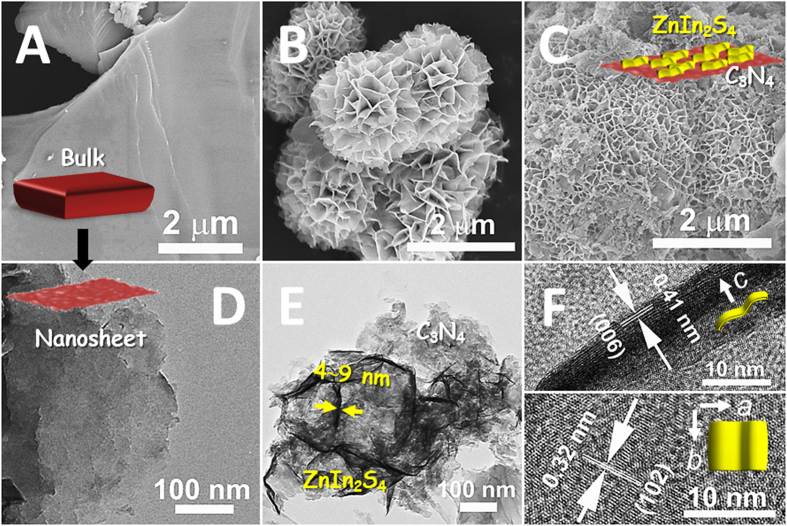
SEM images of (**A**) bulk g-C_3_N_4_, (**B**) ZnIn_2_S_4_ nanosheets, and (**C**) ZnIn_2_S_4_/g-C_3_N_4_ heterojunction nanosheets; TEM images of (**D**) the exfoliated g-C_3_N_4_ nanosheet and (**E**) 15 wt% ZnIn_2_S_4_/g-C_3_N_4_ heterojunction nanosheets; (**F**) HRTEM images of the side view and top view of ZnIn_2_S_4_ nanosheet grown on the g-C_3_N_4_ nanosheets. Insets showing structure schematic diagrams of the corresponding samples.

Scanning electron microscopy (SEM) and transmission electron microscopy (TEM) images were performed to directly observe the morphologies and structures of the ZnIn_2_S_4_/g-C_3_N_4_ heterojunction nanosheets in comparison with the single component of ZnIn_2_S_4_ and g-C_3_N_4_ nanosheets, as displayed in Fig. 2. The thermal polymerized g-C_3_N_4_ shows a bulk structure with smooth surfaces (Fig. 2A), which can be easily exfoliated into the wrinkled sheet-like nanostructures by the ultrasonic treatment in methanol solution (Fig. 2D). Figure 2B reveals significant aggregation of the nanosheets into microspheres with the average diameter of ~1.5 μm for the hydrothermally synthesized ZnIn_2_S_4_ sample. Interestingly, when introducing the as-fabricated g-C_3_N_4_ nanosheets as the substrates during the hydrothermal process, the ZnIn_2_S_4_/g-C_3_N_4_ heterojunction nanosheets could be achieved in the form of “sheet-on-sheet” structure (Figure S2 and Figure S3). As observed in Fig. 2C, the layered surfaces of g-C_3_N_4_ nanosheet are covered with the high density of secondary ZnIn_2_S_4_ nanosheets. These nanosheets with uniformly ultrathin 2D-structure are connected and even across to each other, finally forming the sheet-like networks vertically aligned on the g-C_3_N_4_ nanosheet surface. This kind of unique hierarchical heterostructure (65.9 m^2^ g^−1^) shows much higher specific-surface-area than the general sheet-like structure for pure g-C_3_N_4_ (12.8 m^2^ g^−1^), thereby providing more active sites for the photocatalytic reaction.(Figure S4) Moreover, the interspaces among interweaved ZnIn_2_S_4_ nanosheets on the g-C_3_N_4_ nanosheets may also boost the light-harvesting behavior of this hierarchical heterostructure though the multi-reflection processes on the incident electromagnetic waves^25^,^26^,^29^. TEM image of the ZnIn_2_S_4_/g-C_3_N_4_ heterojunction nanosheets further confirms that the ultrathin ZnIn_2_S_4_ nanosheets with thickness of 4∼9 nm are vertically grown onto the g-C_3_N_4_ nanosheets surface (Fig. 2E and Figure S3). Figure 2F presents the high-resolution (HR) TEM of an individual ZnIn_2_S_4_ nanosheet on g-C_3_N_4_ surface, in which the lattice-fringe spacing of 0.41 nm appeared on the side view (perpendicular sheet) can be assigned to the (006) crystal plane of hexagonal ZnIn_2_S_4_, while the top view (planar sheet) image shows the interplanar distances of 0.32 nm, belonging to the *d*-spacing of (102) planes of hexagonal ZnIn_2_S_4_. In short, intimate contacted heterojunctions between ZnIn_2_S_4_ and g-C_3_N_4_ nanosheets are indeed constructed by the *in-situ* growth process, which may be beneficial for the photoinduced interfacial charge-transfer from g-C_3_N_4_ to ZnIn_2_S_4_.

**Figure 3:**
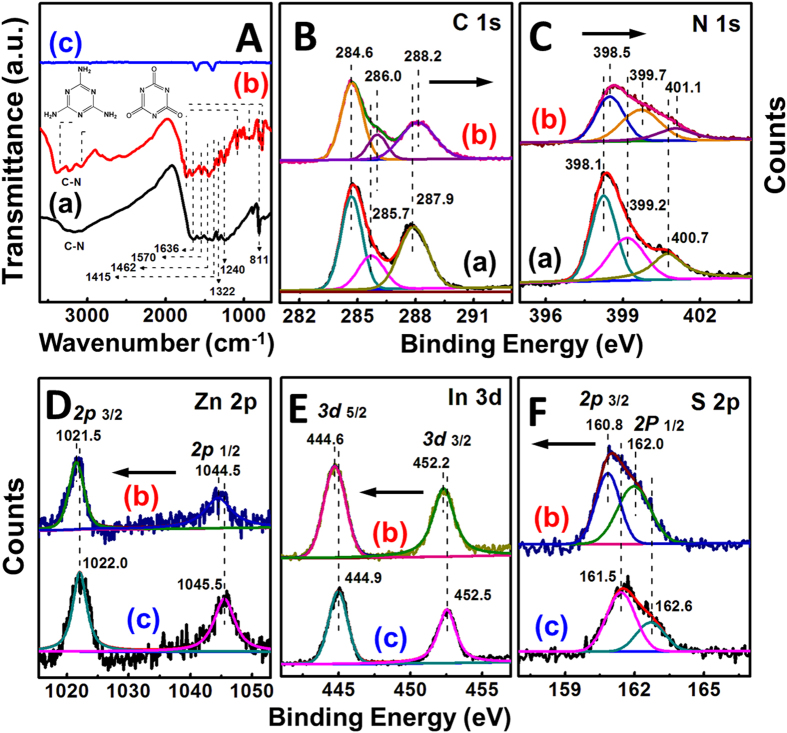
(**A**) FT-IR spectra of the as-synthesized samples: (a) g-C_3_N_4_ nanosheets, (b) 15 wt% ZnIn_2_S_4_/g-C_3_N_4_ heterojunction nanosheets, and (c) ZnIn_2_S_4_ nanosheets; XPS spectra of the as-synthesized samples: (**B**) C 1s core-level spectra; (**C**) N 1s core-level spectra: (a) g-C_3_N_4_ nanosheets, (b) 15 wt% ZnIn_2_S_4_/g-C_3_N_4_ heterojunction nanosheets; (**D**) Zn 2p core-level spectra; (**E**) In 3d core-level spectra; (**F**) S 2p core-level spectra: (b) 15 wt% ZnIn_2_S_4_/g-C_3_N_4_ heterojunction nanosheets, and (c) ZnIn_2_S_4_ nanosheets.

Figure 3A presents the Fourier transform infrared (FT-IR) spectra of the as-synthesized ZnIn_2_S_4_/g-C_3_N_4_ heterojunction nanosheets along with the single ZnIn_2_S_4_ and g-C_3_N_4_ nanosheets for the purpose of structure comparison. The stretching vibration bands on the spectra of g-C_3_N_4_ nanosheets show characteristics similar to those of the reported results^13^,^16^,^21^,^23^. Accordingly, the peaks appeared between 1200 cm^−1^ and 1650 cm^−1^ are attributed to the stretching vibration modes of CN heterocycles. The peak located at 3200 cm^−1^ is originated to the NH stretching vibration mode, while the 811 cm^−1^ to the feature vibration mode of *s*-triazine ring unit. For the ZnIn_2_S_4_ sample, only two peaks at 1396 cm^−1^ and 1610 cm^−1^, belonging to the surface hydroxyl groups and absorbed water molecules, can be observed on the FT-IR spectrum^25^. After the growth of ZnIn_2_S_4_ nanosheets onto g-C_3_N_4_ surfaces, the heterojunction nanosheets show the typical stretching vibration modes of both ZnIn_2_S_4_ and g-C_3_N_4_ nanosheets. Besides, a series of new vibration bands can be detected simultaneously on the spectrum of ZnIn_2_S_4_/g-C_3_N_4_ nanosheets, which are in agreement with the vibration bands of melamine and/or ammeline^16^,^36^. This further suggests that during the long-time hydrothermal process, a few number of g-C_3_N_4_ nanosheets were exfoliated (or reduced) into the sub-structures of g-C_3_N_4_. To study in-depth the chemical configurations of the as-synthesized samples, the X-ray photoelectron spectroscopy (XPS) analyses were performed. As observed in Fig. 3B, three main peaks with the binding energies at 284.6 eV, 285.7 eV, and 287.9 eV can be found on the C 1s core-level spectrum of g-C_3_N_4_ nanosheets, which are assigned to sp^2^ C-C bonds of graphitic carbon, sp^3^-coordinated carbon bonds, and sp^2^-bonded carbon (N-C = N) of the s-triazine rings, respectively^17^,^21^. The binding energy for the C 1s peak at 284.6 eV can be attributed to the adventitious carbon species on the samples and the carbon-containing contaminants, which was used as the reference for calibration. The N 1s signal of g-C_3_N_4_ nanosheets also shows three feature peaks, corresponding to the sp^2^-bonded N (C-N = C) (398.1 eV), tertiary nitrogen N-(C)_3_ groups (399.2 eV), and amino groups (C-N-H) (400.7 eV)^17^,^21^. Investigations found that the relative intensity of the peaks relating to the N-C = N and C-N = C groups of g-C_3_N_4_ nanosheets are decreased after hydrothermal treatment for a long time, indicating that some of the tri-s-triazine units of g-C_3_N_4_ were distorted during this process. Meanwhile, when introducing the ZnIn_2_S_4_ nanosheets onto g-C_3_N_4_ nanosheets to form the heterojunction, both the C 1s and N 1s characteristic signals of g-C_3_N_4_ nanosheets shift slightly toward the higher binding energy side. On the contrary, the binding energies of Zn *2p* (1022.0 eV for *2p*_3/2_ and 1045.5 eV for *2p*_1/2_), In *3d* (444.9 eV for *3d*_5/2_ and 452.5 eV for *3d*_3/2_), and S *2p* (161.5 eV for *2p*_3/2_ and 162.6 eV for *2p*_1/2_) for the ZnIn_2_S_4_/g-C_3_N_4_ heterojunction nanosheets are a little lower than the corresponding values for the pure ZnIn_2_S_4_ nanosheets^25^,^29^,^31^, as shown in Fig. 3D–F. The binding energy shifts λfor the heterojunction components could be explained by a strong interaction between ZnIn_2_S_4_ and g-C_3_N_4_ nanosheets^21^,^37^,^38^,^39^. Theoretically, the enhancement of binding energy means the weakened electron screening effect caused by the decreased electron concentration, while the increase in electron concentration leads to the decrease of binding energy due to the promoted electron screening effect. Thus, in this case, it is reasonable to conclude that the higher and lower binding energy shifts are ascribed to the decreased electron concentration of g-C_3_N_4_ nanosheets and increased electron concentration of ZnIn_2_S_4_ nanosheets due to the strong interaction between the g-C_3_N_4_ to ZnIn_2_S_4_ nanosheets based on the interfacial charge transfer.

**Figure 4:**
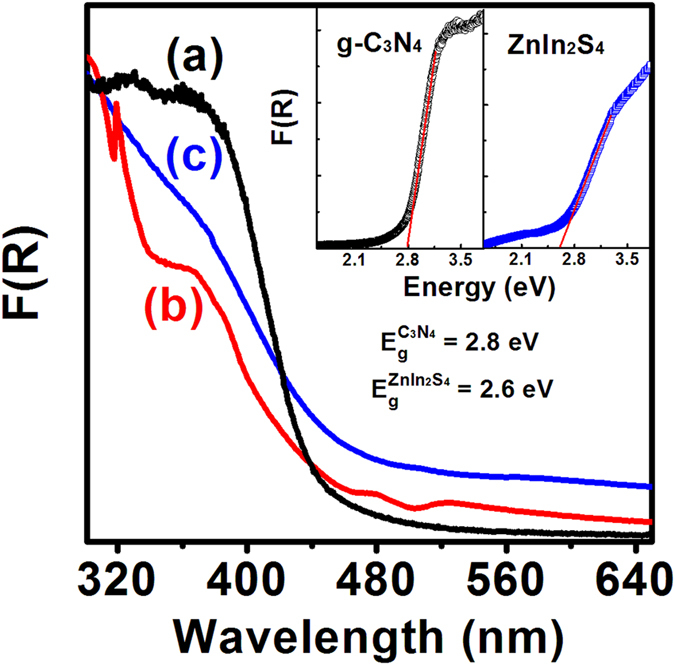
UV-Vis absorption spectra of the as-synthesized samples: (**a**) g-C_3_N_4_ nanosheets, (**b**) 15 wt% ZnIn_2_S_4_/g-C_3_N_4_ heterojunction nanosheets, and (**c**) ZnIn_2_S_4_ nanosheets; insets showing the plots of the F(R) versus energy for the g-C_3_N_4_ and ZnIn_2_S_4_ nanosheets.

The optical properties of the as-synthesized samples were investigated through UV-vis absorption spectra which converted from the corresponding diffuse reflectance (DR) spectra by means of the Kubelka-Munk function. As shown in Fig. 4, the absorption edges of g-C_3_N_4_ and ZnIn_2_S_4_ nanosheets appears at ~443 and ~476 nm, corresponding to the band energies of ~2.8 and ~2.6 eV, respectively. These values are consistent with the reported values of g-C_3_N_4_ and ZnIn_2_S_4_ nanosheets^15^,^17^,^18^,^19^,^28^,^33^,^34^. In the case of ZnIn_2_S_4_/g-C_3_N_4_ heterojunction nanosheets, two obvious absorption bands ascribed to the characteristic absorption of g-C_3_N_4_ and ZnIn_2_S_4_ nanosheets can be found on the absorption curve of Fig. 4b. Moreover, the absorption peaks of ZnIn_2_S_4_ nanosheets become more intense with increase of ZnIn_2_S_4_ content in the heterojunction nanosheets (Figure S5), further confirming that the ZnIn_2_S_4_/g-C_3_N_4_ heterojunction nanosheets with controllable component contents were obtained.

## Discussion

**Figure 5:**
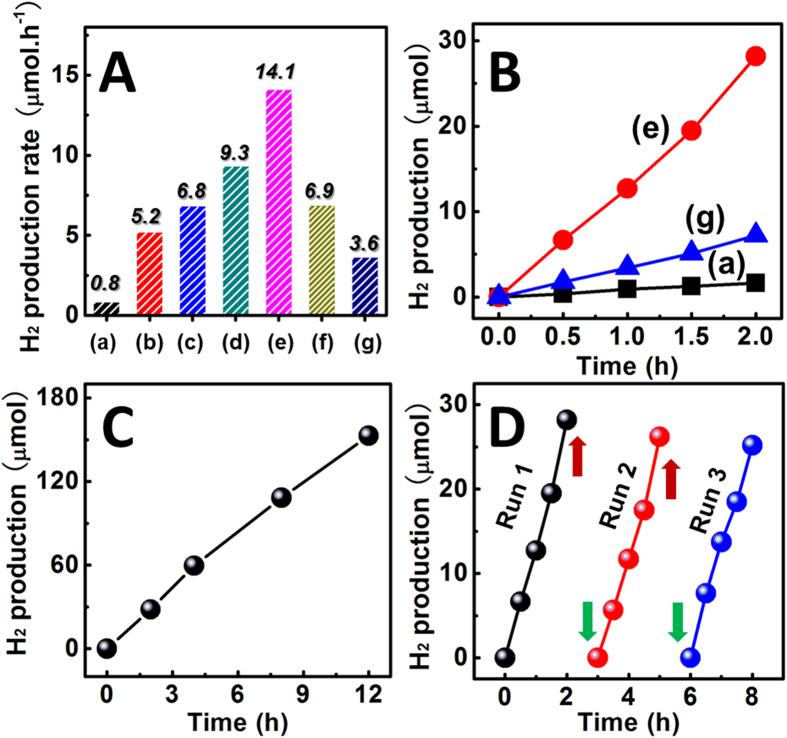
(**A**) Photocatalytic H_2_ production under visible light irradiation over (a) g-C_3_N_4_ nanosheets; (e) 15 wt% ZnIn_2_S_4_/g-C_3_N_4_ heterojunction nanosheets, and (g) ZnIn_2_S_4_ nanosheets; (**B**) comparison of visible-light-driven H_2_ production rate over different samples: (a) g-C_3_N_4_ nanosheets, (b) 2.5 wt%, (c) 5 wt%, (d) 10 wt%, (e) 15 wt%, (f) 20 wt% ZnIn_2_S_4_/g-C_3_N_4_ heterojunction nanosheets, and (g) ZnIn_2_S_4_ nanosheets; (**C**) photocatalytic H_2_ production curve with prolonged irradiation time over 15 wt% ZnIn_2_S_4_/g-C_3_N_4_ heterojunction nanosheets; (**D**) cycling test of photocatalytic H_2_ production over 15 wt% ZnIn_2_S_4_/g-C_3_N_4_ heterojunction nanosheets.

Photocatalytic H_2_ production activities of the as-synthesized samples were evaluated under visible light (λ > 400 nm) irradiation by using triethanolamine (TEOA) as the sacrificial reagent to quench the photoinduced holes. The H_2_ production rates of pure g-C_3_N_4_ nanosheets, pure ZnIn_2_S_4_ nanosheets, and ZnIn_2_S_4_/g-C_3_N_4_ heterojunction nanosheets with various ZnIn_2_S_4_ ratios are summarized in Fig. 5A, in which the heterojunction nanosheets show the enhanced H_2_ production rates as compared to the single heterojunction components. It implies that *in-situ* growth of ZnIn_2_S_4_ nanosheets onto g-C_3_N_4_ nanosheets could noticeably improve the photocatalytic activities on H_2_ production. Even with only 2.5 wt% ZnIn_2_S_4_ nanosheets, the heterojunction nanosheet displays a H_2_ production rate of 5.2 μmol h^−1^, which is more than 6 times higher than that of pure g-C_3_N_4_ nanosheets (0.8 μmol h^−1^). The poor photoactivity of g-C_3_N_4_ nanosheets for the H_2_ production is mainly ascribed to its limited light-harvesting efficiency and fast recombination of photoinduced charge carriers^18^,^19^. The optimal photocatalytic activity was achieved on 15 wt% ZnIn_2_S_4_ with a H_2_ production rate of 14.1 μmol h^−1^. This value is ~17.6 times higher than that of pure g-C_3_N_4_ nanosheets and even nearly 4 times higher that of the pure ZnIn_2_S_4_ nanosheets. Accordingly, the apparent quantum efficiency of this optimal sample is estimated as 0.28% under irradiation at 420 nm. Note that only 5 mg of 15 wt% ZnIn_2_S_4_/g-C_3_N_4_ heterojunction nanosheets was used for H_2_ production in our work. However, when the ZnIn_2_S_4_ content is higher than 15 wt%, a further increase in ZnIn_2_S_4_ content (20 wt%) leads to a rapid decrease in the photocatalytic activity for H_2_ production. This photoactivity reduction can be attributed to the increased opacity (so-called shield effect), resulting in a decrease of irradiation passing through the suspension photoreaction solution^21^,^25^,^29^,^31^,^40^,^41^. As observed in Fig. 5B, the significant enhancement of photocatalytic activity for the heterojunction sample can be further confirmed by the time-dependent H_2_ production behaviors. It could be found that the H_2_ production amounts linearly increases with the irradiation time. After visible light irradiation for 2h, the H_2_ production yield of 15 wt% ZnIn_2_S_4_-decorated g-C_3_N_4_ nanosheet could reach 28.2 μmol, which is greatly superior to the pure g-C_3_N_4_ and ZnIn_2_S_4_ nanosheets. The enhanced photoactivity on H_2_ production could be explained by two main reasons: (1) the reduced recombination process of photoinduced charge carriers on g-C_3_N_4_ and increased amount of charge carriers on ZnIn_2_S_4_ based on the interfacial charge transfer; (2) the higher specific-surface-area and enhanced light absorption for the unique “sheet-on-sheet” heterostructure as aforementioned. However, it should be point out that in comparison with the pure ZnIn_2_S_4_ nanosheets (132.0 m^2^ g^−1^), the heterojunction nanosheets (65.9 m^2^ g^−1^) shows the lower specific-surface-area, but the higher photoactivity. Meanwhile, the g-C_3_N_4_ nanosheets treated by the hydrothermal process in the absence of ZnIn_2_S_4_ precursor show a lower photocatalytic H_2_ production rate (0.41 μmol/h) as compared to the pure (untreated) g-C_3_N_4_ nanosheets (Figure S6), because of the poor photoactivities of the exfoliation or reduction of g-C_3_N_4_ with the ultra-small structures, such as melamine, ammeline, or tri-s-triazine units^16^,^36^. These observations also the indirect evidence that the dynamics process of photoinduced charge transfer occurring on the interface between the ZnIn_2_S_4_ and g-C_3_N_4_ nanosheets may be crucial to the photocatalytic H_2_ production activities of heterojunction nanosheets. At low ZnIn_2_S_4_ content, a well dispersion of ZnIn_2_S_4_ nanosheets on the g-C_3_N_4_ nanosheets could be formed. In this way, the increase of ZnIn_2_S_4_ content would induce more ZnIn_2_S_4_ nanosheets assembled onto the g-C_3_N_4_ surface, which generates larger contact area between the ZnIn_2_S_4_ and g-C_3_N_4_ nanosheets, thereby allowing more efficient interfacial charge transfer. However, excess ZnIn_2_S_4_ loading causes considerable reduction of light absorption for the covered g-C_3_N_4_, and decreases its excitation process for interfacial charge transfer. Therefore, a balance would be built between ZnIn_2_S_4_ loading amount and ZnIn_2_S_4_/g-C_3_N_4_ contact area, which can maximize the photocatalytic H_2_ production for the heterojunction nanosheets. Through the sequential remediation of heterojunction components, we concluded an optimal loading of 15 wt% ZnIn_2_S_4_ onto g-C_3_N_4_, which exhibited the highest photocatalytic activity in our work.

Besides, this optimal ZnIn_2_S_4_/g-C_3_N_4_ heterojunction nanosheets also shows fairly stable photoactivity for H_2_ production. In Fig. 5C, the H_2_ production rate remains consistent even at the prolonged time period for 12 h. Meanwhile, the recycling ability for the 15 wt% ZnIn_2_S_4_/g-C_3_N_4_ heterojunction nanosheets was further studied by performing a three-run test of photocatalytic H_2_ production. Figure 5D shows that no notable decreases of H_2_ evolution were detected during the three-run test, powerfully verifying the good stability of 15 wt% ZnIn_2_S_4_/g-C_3_N_4_ heterojunction nanosheets for using as the photocatalysts.

**Figure 6:**
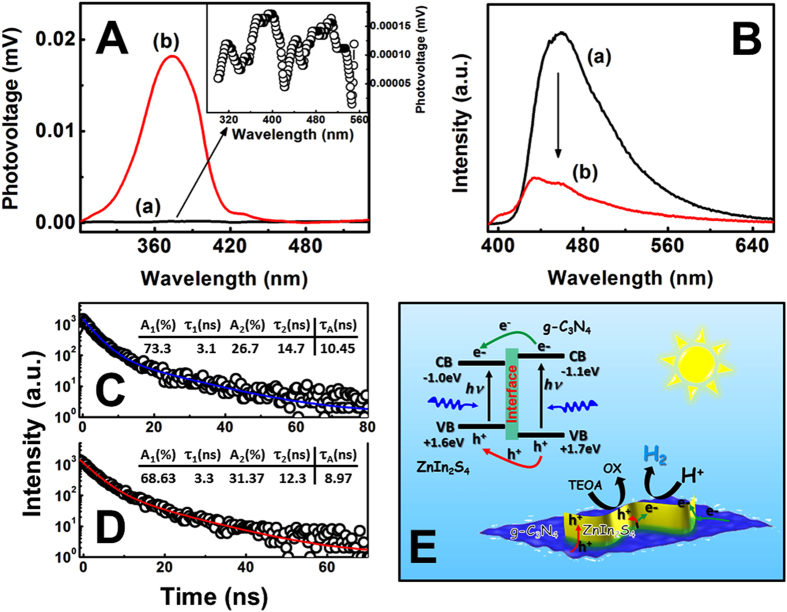
(**A**) SPS of the as-synthesized samples: (a) g-C_3_N_4_ nanosheets, (b) 15 wt% ZnIn_2_S_4_/g-C_3_N_4_ heterojunction nanosheets; (**B**) steady-state PL spectra of (a) g-C_3_N_4_ nanosheets and (b) 15 wt% ZnIn_2_S_4_/g-C_3_N_4_ heterojunction nanosheets; Time-resolved transient PL decay of (**C**) g-C_3_N_4_ nanosheets and (**D**) 15 wt% ZnIn_2_S_4_/g-C_3_N_4_ heterojunction nanosheets; (**E**) schematic diagram showing the photoinduced charge transfer in the interface between ZnIn_2_S_4_ and g-C_3_N_4_ nanosheets.

To provide more evidence on the photoinduced charge transfer occurring in the heterojunction interface, the surface photovoltage spectroscopy (SPS) and photoluminescence (PL) spectroscopy of ZnIn_2_S_4_/g-C_3_N_4_ heterojunction nanosheets were investigated in comparison with those of pure g-C_3_N_4_ nanosheets. As shown in the inset of Fig. 6A, a poor response appears on the SPS curve of pure g-C_3_N_4_ nanosheets, indicating very low efficiency on the photovoltaic conversion. However, the photon absorption and conversion processes have been proven by the photoelectrochemistry in our previous work^42^. Thus, the low photovoltage of g-C_3_N_4_ nanosheets can be attributed to the Schottky interface contact between the g-C_3_N_4_ and ITO glass in the absence of electrolyte, which leads to the limited electron transfer process from the g-C_3_N_4_ to ITO glass electrode. After decoration of ZnIn_2_S_4_ onto g-C_3_N_4_ nanosheets, the SPS signal in the region from 300 to 450 nm is remarkably enlarged, suggesting the promoted charge generation and separation based on the semiconductor heterojunction effect. This phenomenon can be further understood through the steady-state and transient PL spectroscopy. In Figre 6B, the pure g-C_3_N_4_ nanosheet shows a strong emission peak with the center at ~450 nm. However, as compared to the g-C_3_N_4_ nanosheets, the emission process of ZnIn_2_S_4_/g-C_3_N_4_ heterojunction nanosheets suppresses significantly, revealing either the faster migration process with the shorter lifetime or the slower recombination process with the longer lifetime for the photoinduced charge carriers. To shed more light on this issue, we tried to fit the time-resolved transient PL spectroscopy based on the multi-exponential kinetics function expressed as follow^43^:


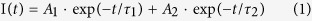


where 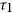 and 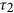 are the fluorescent lifetime, and A_1_, and A_2_ are the corresponding amplitudes. As listed in the insets of Fig. 6C,D, the short lifetime component for 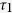 is originated from the nonradiative recombination of charge-carriers in the defect states of g-C_3_N_4_, while the longer lifetime component for 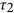 is caused by the free excitons recombination in the g-C_3_N_4_ body^12^,^21^,^34^,^42^. In the case of ZnIn_2_S_4_/g-C_3_N_4_ heterojunction nanosheets, the emission lifetime for the component 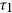
(3.3 ns) is longer than the corresponding lifetime of g-C_3_N_4_ nanosheets (3.1 ns), while its component 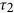 (12.3 ns) is shorter than the component for g-C_3_N_4_ nanosheets (14.7 ns). To gain further understanding on this phenomenon, the average emission lifetimes, relating to the overall emission decay behaviors of the samples, were also evaluated through the following equation^44^:


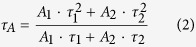


It is clearly that after loading the ZnIn_2_S_4_ nanosheets, the average lifetime of g-C_3_N_4_ nanosheets is shorten from 10.45 ns to 8.97 ns. The combination of decreased emission lifetime and quenched PL indicates the emergence of a nonradiative pathway from the electron transfer between ZnIn_2_S_4_ and g-C_3_N_4_ nanosheets^45^. According to the energy band structures of these semiconductors (Fig. 6E), it could be deduced that the photoinduced electrons transfer from the conduction band (CB) of g-C_3_N_4_ to the CB of ZnIn_2_S_4_ nanosheets. This assumption is in agreement with the emission quenching phenomenon of g-C_3_N_4_ nanosheets after decorating the ZnIn_2_S_4_ nanosheets. The rate constant for the interfacial electron-transfer 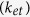 can be estimated by the expression^46^:


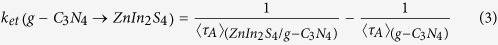


Obviously, the 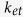 value is approximately 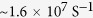. As illustrated in Fig. 6E, the CB and valence band (VB) positions of g-C_3_N_4_ straddle those of ZnIn_2_S_4_, forming the “type I” heterojunction interface. When this heterojunction is excited by visible light with the photon energy higher or equal to the band gaps of both ZnIn_2_S_4_ and g-C_3_N_4_ nanosheets, the photoinduced electrons and holes of g-C_3_N_4_ nanosheets would move to the CB and VB of ZnIn_2_S_4_ nanosheets, respectively. As such, the recombination process on the photoinduced charge carriers of g-C_3_N_4_ could be suppressed effectively by the photosynergistic effect of ZnIn_2_S_4_/g-C_3_N_4_ heterojunction. Accordingly, the amount of photoinduced charge carriers on ZnIn_2_S_4_ is remarkably increased based on the photoinduced interfacial charge transfer. During the photocatalytic H_2_ production process, the photoinduced electrons accumulated on the CB of ZnIn_2_S_4_ could initiate the catalytic proton reduction to H_2_. Accordingly, the photoinduced holes transfer from the VB of g-C_3_N_4_ to the VB of ZnIn_2_S_4_ were quenched by the sacrificial reagent of TEOA (or directly quenched by the sacrificial reagent on the VB of g-C_3_N_4_). In this way, the effective charge transfer at the interface between the ZnIn_2_S_4_ and g-C_3_N_4_ nanosheets results in the enhanced photocatalytic activity on H_2_ production.

In summary, a series of ZnIn_2_S_4_/g-C_3_N_4_ heterojunction nanosheets with various contents of ZnIn_2_S_4_ have been successfully synthesized through *in-situ* growth of ultrathin ZnIn_2_S_4_ nanosheets onto g-C_3_N_4_ nanosheets fabricated by a traditional thermal polymerization and followed ultrasonic dispersion method. The unique “sheet-on-sheet” heterostructure obtained by vertically loading ZnIn_2_S_4_ nanosheets onto the g-C_3_N_4_ nanosheets surfaces leads to the enlarged reactive sites and enhanced light absorption ability. More importantly, the formation of “type I” heterojunction can effectively suppress the photoinduced charge recombination of g-C_3_N_4_ through the interfacial charge transfer, as evidenced by the electron microscopic analyses, steady-state and time-resolved transient photoluminescence decay investigations. As a result, the ZnIn_2_S_4_/g-C_3_N_4_ heterojunction nanosheets exhibited considerable enhancement on the photocatalytic activity for H_2_ production as compared the single component nanosheets. It is believed that our study provides a promising strategy to develop the new generation of hierarchical heterostructure photocatalysts for highly efficient solar-to-fuels conversion and environmental remediation.

## Methods

### Materials synthesis

The graphitic carbon nitride (g-C_3_N_4_) was obtained by a traditional thermal polymerization method. 10 g of melamine powder was grinded for 60 min in a mortar and then transferred to an alumina crucible with a cover. Afterward, the crucible was heated to 550 °C with a rising rate of 20 °C min^−1^ and kept for 2 h at the required temperature under semiclosed environment, resulting in the bulk g-C_3_N_4_ with faint-yellow color. ZnIn_2_S_4_/g-C_3_N_4_ heterojunction nanosheets were synthesized by *in-situ* growth of ultrathin ZnIn_2_S_4_ nanosheets onto g-C_3_N_4_ nanosheets through a facile hydrothermal method. In a typical procedure, 600 mg of as-synthesized bulk g-C_3_N_4_ was grinded to fine powder and then added into 20 ml of methanol. After ultrasonic treatment for 2 h, the bulk g-C_3_N_4_ was exfoliated into thin nanosheets which was then collected and washed by using centrifugation-redispersion with deionized water. Subsequently, these exfoliated g-C_3_N_4_ nanosheets was resuspended into 20 ml of premade aqueous solution consisting of 0.2125 mmol of Zn(CH_3_COO)_2_ · 2H_2_O, 0.425 mmol of In(NO_3_)_3_ · 6H_2_O, and 1.7 mmol of L-cysteine. After being ultrasonically treated for 30 min, this mixture was transferred into a Teflon-lined stainless steel autoclave with a capacity of 25 mL. Afterward, the autoclave was sealed and maintained at 180 °C for 12 h in an electric oven. When natural cooling the autoclave to room temperature, the yellow-green suspension was collected, washed with ethanol and deionized water for several times, and finally dried in an electric oven at 60 °C for a night. Thus, the 15 wt% ZnIn_2_S_4_/g-C_3_N_4_ heterojunction nanosheets were synthesized. The pure ZnIn_2_S_4_ nanosheets were fabricated by the same hydrothermal conditions in the absence of the g-C_3_N_4_ nanosheets substrates. Meanwhile, to achieve the optimal photocatalytic activity, the ZnIn_2_S_4_/g-C_3_N_4_ heterojunction nanosheets with different ZnIn_2_S_4_ loading amount were also synthesized using the similar route by adjusting the concentrations of hydrothermal precursor solution in the same component ratios. In order to further prove that the enhanced photocatalytic activity of ZnIn_2_S_4_/g-C_3_N_4_ nanosheets is due to the heterojunction effect, another control sample were fabricated through hydrothermal treatment of pure g-C_3_N_4_ nanosheets in the absence of the above ZnIn_2_S_4_ precursors.

### Characterization methods

X-ray diffraction (XRD) patterns of the as-synthesized samples were measured by a Shimadzu XRD-6000 X-ray diffractometer with a Cu Kα line of 0.1541 nm. Scanning electron microscopy (SEM; XL-30 ESEM FEG, Micro FEI Philips) and transmission electron microscopy (TEM; JEOL JEM-2100) were employed to observe the morphologies and structures of the samples. Energy dispersive X-ray (EDX) spectroscopy being attached to scanning electron microscopy (SEM) was used to analyze the composition of products. Fourier transform infrared (FT-IR) spectra were recorded on a Magna 560 FT-IR spectrometer with a resolution of 1 cm^−1^. X-ray photoelectron spectroscopy (XPS) was carried out on a VG-ESCALAB LKII instrument with a Mg Kα ADES (*hν* = 1253.6 eV) source at a residual gas pressure below 10^−8^ Pa. UV-vis diffuse reflectance spectra (DRS) were taken with a Lambda 750 UV/Vis/NIR spectrophotometer (Perkin Elmer, USA). The specific surface areas of the products were measured with a Micromeritics ASAP-2020 instrument and analyzed by the Brunauer–Emmett–Teller (BET) method. Decay curves of the as-fabricated products were obtained on a FLS920 fluorescence lifetime spectrophotometer (Edinburgh Instruments, UK) under the excitation of a hydrogen flash lamp with the wavelength at 325 nm (nF900; Edinburgh Instruments). The surface photovoltage spectroscopy (SPS) was performed on PL-SPS1000 instrument (Beijing Perfectlight Technology Co., Ltd). During the process, the sample was put between the indium tin oxide (ITO) glass and stainless steel electrodes to form a sandwich structured photovoltage cell.

### Photocatalytic H_2_ production

The photocatalytic H_2_ production tests were performed in a 35-mL quartz reactor. Typically, 5 mg of the as-synthesized samples were suspended in 10-mL triethanolamine (TEOA, 15 vol.%) aqueous solution. This suspension was sealed in the quartz reactor by a rubber plug, and then purged with argon gas for half an hour to drive away the residual air. Subsequently, the reactor was exposed under a 300-W Xe lamp (PLS-SXE300UV) coupled with a 400 nm cut-off filter. The gas product composition from the upper space above the liquid in the quartz reactor was periodically analyzed by a gas chromatograph (GC) equipped with a thermal conductivity detector (TCD) (Beifen-Ruili Analytical Instrument, SP-3420A). The apparent quantum efficiency (QE) was estimated by using the following equation.


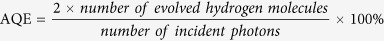


## Additional Information

**How to cite this article**: Zhang, Z. *et al.* Hierarchical Sheet-on-Sheet ZnIn_2_S_4_/g-C_3_N_4_ Heterostructure with Highly Efficient Photocatalytic H_2_ production Based on Photoinduced Interfacial Charge Transfer. *Sci. Rep.* **6**, 19221; doi: 10.1038/srep19221 (2016).

## Supplementary Material

Supporting Information
