# Supplementary material for: Hierarchical Sheet-on-Sheet ZnIn2S4/g-C3N4 Heterostructure with Highly Efficient Photocatalytic H2 production Based on Photoinduced Interfacial Charge Transfer
Source: Sci Rep. 2016 Jan 12;6:19221. doi: 10.1038/srep19221 (PMC4709776; doi:10.1038/srep19221)
Supplement: Supporting Information [file srep19221-s1.doc]

**Supplementary Information**

**Hierarchical Sheet-on-Sheet ZnIn_2_S_4_/g-C_3_N_4_ Heterostructure with Highly Efficient Photocatalytic H_2_ production Based on** **Photoinduced Interfacial Charge Transfer**

Zhenyi Zhang, Kuichao Liu, Zhiqing Feng, Yanan Bao, Bin Dong*^*^*

*Key Laboratory of New Energy and Rare Earth Resource Utilization of State Ethnic Affairs Commission,* *School of Physics and Materials Engineering, Dalian Nationalities University, 18 Liaohe West Road, Dalian 116600, P. R. China*

* Corresponding author:

Key Laboratory of New Energy and Rare Earth Resource Utilization of State Ethnic Affairs Commission, School of Physics and Materials Engineering, Dalian Nationalities University, 18 Liaohe West Road, Dalian 116600, P. R. China.

E–mail: [dong@dlnu.edu.cn](mailto:dong@dlnu.edu.cn); Tel: +86 41187556959.


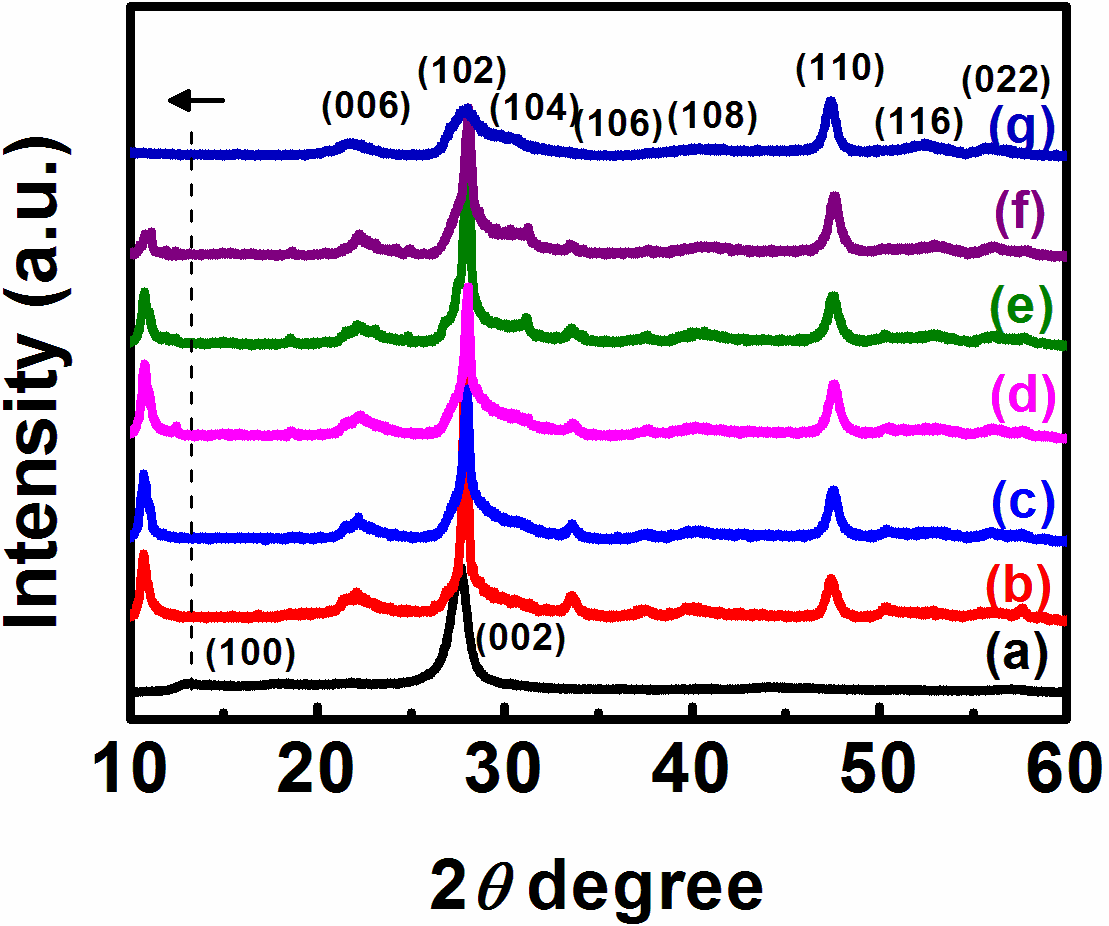


**Figure S1** XRD patterns of the as-synthesized samples: (a) g-C_3_N_4_ nanosheets, (b) 2.5 wt%, (c) 5 wt%, (d) 10 wt%, (e) 15 wt%, (f) 20 wt% ZnIn_2_S_4_/g-C_3_N_4_ heterojunction nanosheets, and (g) ZnIn_2_S_4_ nanosheets.


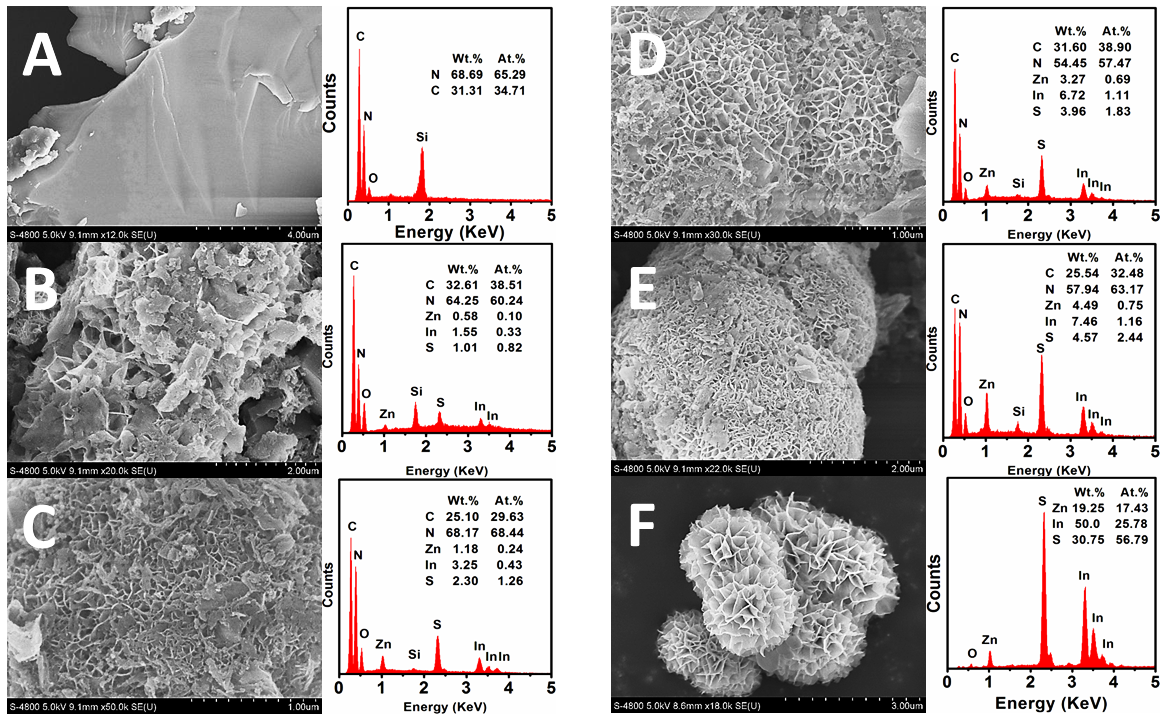


**Figure S2** SEM and the corresponding energy-dispersive X-ray (EDX) spectra of the as-synthesized nanosheets: (A) g-C_3_N_4_ nanosheets; (B) 2.5 wt%; (C) 5 wt%; (D) 15 wt%; (E) 20 wt% ZnIn_2_S_4_/g-C_3_N_4_ heterojunction nanosheets; (F) ZnIn_2_S_4_ nanosheets.

The EDX results show that the tested weight percentages of ZnIn_2_S_4_ to g-C_3_N_4_ in the heterojunction nanosheets are 3.2 wt.%, 7.2 wt.%, 16.2 wt.% and 19.8 wt.% for the samples named as the 2.5 wt.%, 5.0 wt.%, 15.0 wt.% and 20.0 wt.% ZnIn_2_S_4_/g-C_3_N_4_ heterojunction nanosheets, respectively. These ratio values are a little different to the theoretical values due to the limited test areas from the SEM images. However, note that the weight percentages of ZnIn_2_S_4_ in the heterojunction nanosheets still gradually increase with the amount of ZnIn_2_S_4_ precursor used in our work.


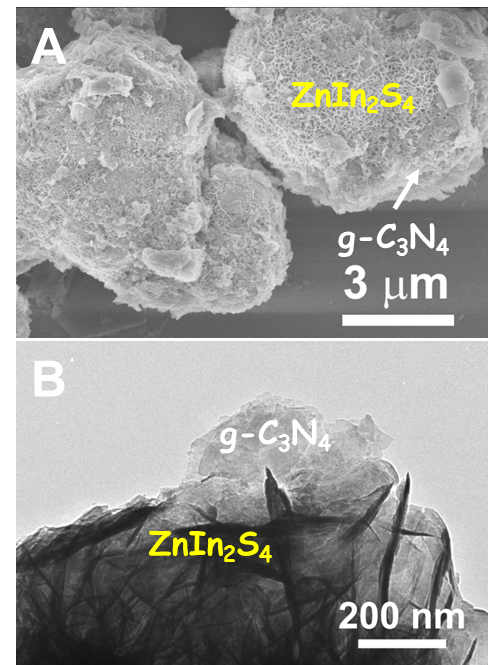


**Figure S3** SEM and TEM images of ZnIn_2_S_4_/g-C_3_N_4_ heterojunction nanosheets showing the “sheet-on-sheet” hierarchical structure.


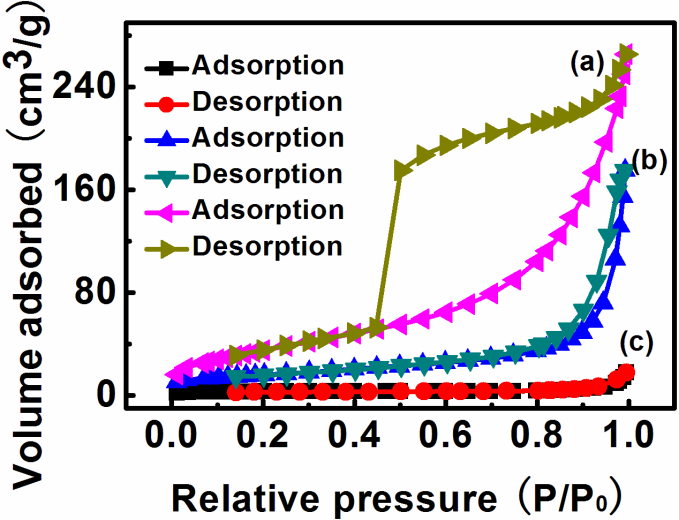


**Figure S4** Nitrogen adsorption-desorption isotherm of the as-synthesized samples: (a) ZnIn_2_S_4_ nanosheets; (b) ZnIn_2_S_4_/g-C_3_N_4_ heterojunction nanosheets; (c) g-C_3_N_4_ nanosheets.


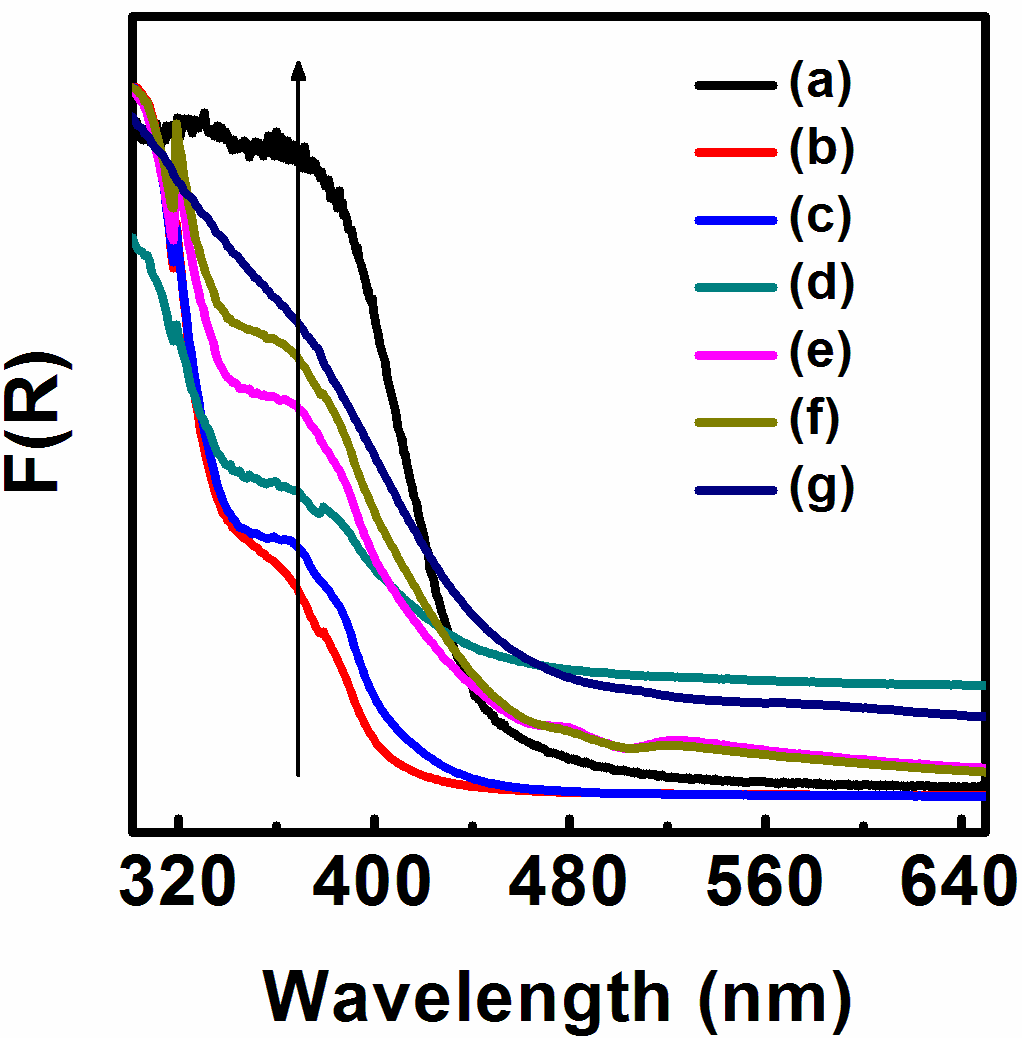


**Figure S5** UV-Vis absorption spectra of the as-synthesized nanosheets, which are converted from diffuse reflectance spectra by means of the Kubelka-Munk function: (a) g-C_3_N_4_ nanosheets, (b) 2.5 wt%, (c) 5 wt%, (d) 10 wt%, (e) 15 wt%, (f) 20 wt% ZnIn_2_S_4_/g-C_3_N_4_ heterojunction nanosheets, and (g) ZnIn_2_S_4_ nanosheets.


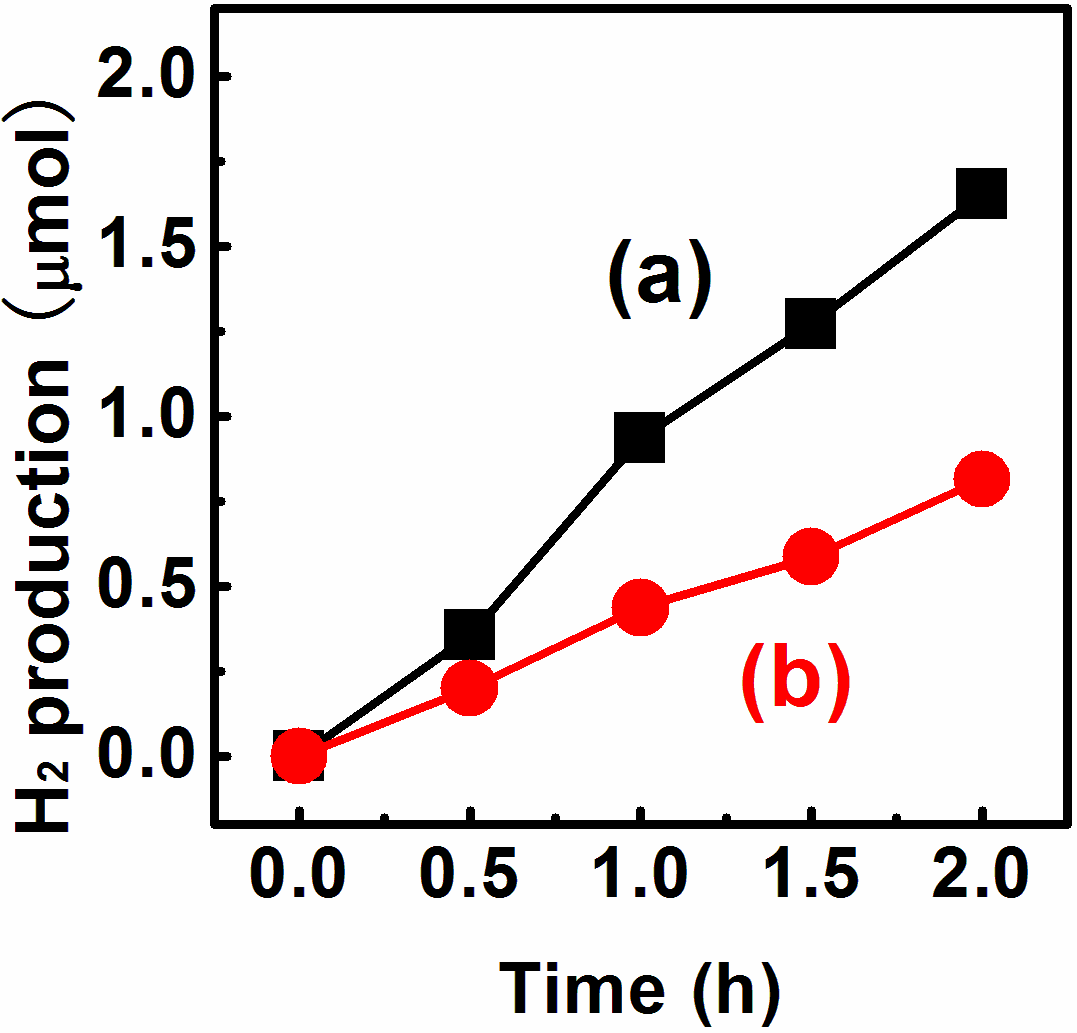


**Figure S6** Comparison of visible-light-driven H_2_ production rate over different samples: (a) pure g-C_3_N_4_ nanosheets; (b) the g-C_3_N_4_ nanosheets treated by the hydrothermal method in the absence of ZnIn_2_S_4_ precursors.
